# NAD+ Suppresses EV-D68 Infection by Enhancing Anti-Viral Effect of SIRT1

**DOI:** 10.3390/v17020175

**Published:** 2025-01-26

**Authors:** Yue Wang, Haiyu Li, Xia Huang, Yan Huang, Mingqi Lv, Hong Tang, Xinyue Han, Juntong Liu, Yan Liang, Guangchao Zang, Nan Lu, Guangyuan Zhang

**Affiliations:** 1 Pathogen Biology and Immunology Laboratory, Lab Teaching & Management Center, School of Basic Medical Sciences, Chongqing Medical University, Chongqing 400016, China; yuewangyuewang@163.com (Y.W.); xiahuang2002@163.com (X.H.); 17880207807@163.com (Y.H.); 190429@cqmu.edu.cn (M.L.); 2023220847@stu.cqmu.edu.cn (X.H.); liujuntong@stu.cqmu.edu.cn (J.L.); ly121584372@163.com (Y.L.); zangguangchao@cqmu.edu.cn (G.Z.); 2 Institute of Intelligent Traditional Chinese Medicine, Chongqing University of Chinese Medicine, Chongqing 402760, China; lihaiyu@cqctcm.edu.cn; 3 Second Affiliated Hospital, Army Medical University, Chongqing 400037, China; tang851017@163.com

**Keywords:** SIRT1, NAD+, EV-D68, replication, metabolism

## Abstract

Enterovirus 68 (EV-D68) is a non-enveloped virus with a positive-sense single-stranded RNA genome that causes respiratory diseases and acute flaccid myelitis, posing significant threats to human health. However, an effective vaccine remains undeveloped. SIRT1, a nicotinamide adenine dinucleotide (NAD+)-dependent enzyme, plays a key role in cellular metabolism, but its interaction with NAD+ during viral infections is not well understood. In this study, through a metabolomics analysis, we demonstrate that EV-D68 infection influences cellular metabolism. Additionally, we show that NAD+ inhibits EV-D68 infection both in vivo and in vitro. EV-D68 reduces cellular NAD+ levels by regulating the expression of enzymes involved in NAD+ consumption and synthesis. Moreover, the infection increases the expression of sirtuin 1 (SIRT1), which inhibits EV-D68 replication in turn. Mechanistically, SIRT1 suppresses EV-D68 5′UTR-mediated translation, and the antiviral effect of SIRT1 on EV-D68 replication is enhanced by NAD+. Collectively, our findings highlight the critical role of NAD+ metabolism in EV-D68 infection and reveal the antiviral potential of SIRT1, providing valuable insights for the development of antiviral strategies.

## 1. Introduction

Enterovirus D68 (EV-D68), a non-enveloped virus with a positive-sense single-stranded RNA genome, belongs to the *Enterovirus* genus within the *Picornaviridae* family. It primarily infects the upper respiratory tract, causing outbreaks of asthma-like respiratory diseases and clusters of cases of acute flaccid myelitis (AFM), a paralytic polio-like condition [1]. These impacts underscore the importance of understanding the mechanisms underlying EV-D68’s pathogenesis and developing effective therapeutic strategies.

Metabolomics is a powerful tool for assessing the global metabolic response of cells to external stimuli by detecting the levels of low-molecular-weight metabolites in real-time processes [2]. Viruses, which lack their own metabolic machinery and enzyme systems, are intracellular parasites that rely on host cells to replicate [3]. Various viruses, including HBV, HSV-1, DENV, and ZIKV, have been shown to hijack the host cell metabolism, reprogramming it to promote viral replication [4,5,6,7]. These metabolic alterations involve pathways such as glycolysis, lipid synthesis, nucleotide biosynthesis, and glutamine metabolism, which vary between different viruses. To effectively combat viral infections, cells must carefully manage the metabolic changes induced by the virus, though this process is not yet fully understood. Furthermore, targeting viral-induced metabolic pathways presents a novel strategy for reducing viral threats. For example, the use of difluoromethylornithine (DFMO) to inhibit polyamine biosynthesis has been shown to negatively affect the replication of various RNA viruses, including VSV, ZIKV, and EV-A71 [8]. Therefore, understanding how viral proteins interact with the host cell machinery to induce widespread metabolic alterations, and elucidating how cells mediate viral clearance, is crucial for elucidating the mechanisms of virus–host interactions and providing new insights into the therapeutic strategies aimed at modulating viral activity.

Nicotinamide adenine dinucleotide (NAD+) is a vital coenzyme and substrate in all living cells, playing a pivotal role in energy metabolism, immunoinflammatory responses, and adaptation to oxidative stress. It serves as an essential component for various enzymes, including sirtuins, poly (ADP-ribose) polymerases (PARPs), and cluster of differentiation 38 (CD38) [9]. Emerging evidence indicates that viral infections can lead to a significant depletion of NAD+ levels [10,11]. Consequently, pharmacological strategies to sustain cellular NAD+ levels may provide a promising approach for preventing certain viral-associated diseases. For instance, supplementation with NAD+ or its precursors has been shown to enhance the activity of antiviral PARP isoforms and inhibit the replication of coronaviruses in vitro [12]. While NAD+ supplementation does not prevent Zika virus replication, it protects Zika virus-infected cells from cell death [10]. The sirtuin family consists of seven members (SIRT1 to SIRT7), with SIRT1 being a key regulator of various cellular pathways, including energy metabolism, stress responses, senescence, cancer, and longevity [13]. And it features a highly conserved catalytic domain and belongs to class III NAD+-dependent histone deacetylases (HDACs) [14]. SIRT1 has been shown to mitigate the accumulation of reactive oxygen species (ROS) following spinal cord injury, thereby protecting the blood–spinal cord barrier (BBB) and promoting repair [15]. Additionally, SIRT1 plays a critical role in modulating the innate immune response by regulating the deacetylation of interferon regulatory factors 3 and 7 (IRF3/7), which are essential for type I interferon (IFN-I) production and maintaining immune system homeostasis [16].

In this study, we demonstrate significant changes in the metabolite profiles following EV-D68 infection and uncover the antiviral effects of NAD+ both in vivo and in vitro by identifying these altered metabolites. EV-D68 infection modulates the expression of NAD+-consuming and NAD+-synthesizing enzymes, leading to a reduction in cellular NAD+ levels. Furthermore, we observed that viral infection induces the expression of SIRT1, which inhibits EV-D68 replication by suppressing 5′-UTR-mediated translation, an effect that is further enhanced by NAD+ supplementation.

## 2. Materials and Methods

### 2.1. Cells, Virus Strains, and Reagents

Human rhabdomyosarcoma (RD), 293T, and A549 cell lines were kept in our laboratory. The media were supplemented with 10% fetal bovine serum (FBS, BI), 1% penicillin/streptomycin (HyClone, Logan, UT, USA), and modified Dulbecco Eagle medium (DMEM, GIBCO), cultured at 37 °C with 5% CO_2_ in a humidity incubator. EV-D68 Fermon (ATCC VR-1826) for cells and EV-D68(ATCC VR-1824) for mice models were kept in our lab and propagated in RD cells.

The compounds and reagents, including NAD+ (HY-B0445), L-Cystathionine (HY-W009749), 8-Hydroxyguanosine (HY-113262), Resveratrol (HY-16561), and Sirtinol (HY-12315), were purchased from MedChem Express (Shanghai, China). The NAD+/NADH Assay Kit with WST-8 (S0175) was purchased from Beyotime (Shanghai, China). Flavin adenine dinucleotide (GC13392) and L-Acetylcarnitine with hydrochloride (GC13314) were obtained from GLPBIO (Montclair, CA, USA).

### 2.2. Virus Infection and TCID50

RD, A549, or 293T cells were seeded in 6-well plates. Upon reaching a cell density of 70–80%, the cells were inoculated with EV-D68 and incubated at 37 °C with 5% CO_2_. After 1.5 h, the medium was replaced by DMEM containing 4% FBS. Cell samples were collected at the relevant hours based on the various experimental requirements. The viral titers of EV-D68 were determined through 50% tissue culture infectious dose (TCID50) assays with the Reed–Muench formula.

### 2.3. Plasmid Construction and siRNA

To generate overexpressed plasmid pBudCE4.1-Sirt1, total RNA was extracted from RD cells using Trizol and reverse-transcribed using the PrimeScript kit (Takara, Osaka, Japan). Thereafter, wild Sirt1 fragments were amplified using SIRT1F and SIRT1R primers and inserted into the SalI and XbaI sites of pBudCE4.1. The plasmid pBudCE4.1-mtSirt1, containing an NLS-mutated Sirt1 gene, was generated by amplifying pBudCE4.1-Sirt1 using primer pairs (Sirt1mt1R/Sirt1mt1F and Sirt1mt2R/Sirt1mt2F; Supplementary Tables S1–S4) and ligated using the Golden Gate one-step method. Amplified fragments were mixed with enzymes, SapI and T4 ligase, in a T4 buffer and incubated at 37 °C for 4 h, after which they were transformed into DH5α competent cells. Colonies were identified by PCR and Sanger sequencing.

To generate the dual-luciferase reporter plasmid (p5Ferm), the 5′UTR region and the first 15 amino acid coding regions in CDs of the EV-D68 Fermon strain were generated. The specific details are as described in prior studies [17]. Sequences of small interfering RNAs (siRNAs) against Sirt1 (si-Sirt1-1, si-Sirt1-2, si-Sirt1-3) and siRNAs (si-NC) were synthesized by Sangon Biotech (Shanghai, China) and are shown in the Supplementary Tables S1–S4.

### 2.4. Western Blot

Mouse pup tissues or cells were lysed using TNE buffer (a 50 mM Tris-Cl [pH 7.4], 150 mM NaCl, 2 mM EDTA [pH 8.0], 0.1% 2-mercapto-ethanol and protease inhibitor cocktail). Samples were heated at 100 °C for 10 min in loading buffer. Then the samples were subjected to a 10% sodium dodecyl sulfate–polyacrylamide gel and transferred to a PVDF membrane. The membrane was blocked for 30 min at room temperature using PBS containing Tween 20 (1/1000) and dissolved skim milk. After that, the PVDF membrane was incubated with the primary antibody for 1.5 h, followed by the secondary antibody for 45 min. The primary antibodies used were mouse anti-EV-D68 VP1 (1:2000, GeneTex, San Antonio, TX, USA), rabbit anti-NAMPT (1:500, Sangon, China), rabbit anti-SIRT1 (1:2000, GeneTex, San Antonio, TX, USA), rabbit anti-NMANT2 (1:2000, Sangon, China), rabbit anti-CD38 (1:2000, Sangon, China), rabbit anti-SIRT2 (1:2000, MCE, China), and rabbit anti-beta actin (1:2000, Proteintech, China). HRP-conjugated goat anti-mouse IgG (1:5000, Sangon, China) and HRP-conjugated goat anti-rabbit IgG (1:5000, Sangon, China) were used as secondary antibodies.

### 2.5. RNA Isolation and Quantitative Real-Time PCR

Total RNA from cells or mouse pup tissues was extracted using TRIzol Reagent (LEAGENE, Beijing, China). The PrimeScriptTM RT kit with gDNA eraser (Takara, Osaka, Japan) was used for the reverse transcription of total RNA; reactions were initiated with 1000 ng of RNA for each sample. The relative gene expression level was determined using BioRun ChemoHS qPCR Mix (SYBR) (Biorun, Wuhan, China). Gene expression changes were calculated by the 2−ΔΔCT method. Glyceraldehyde-3phosphate dehydrogenase (GAPDH) was applied as the internal reference to normalize the target gene expression. All the primer sequences used are described in the Supplementary Tables S1–S4 (Tsingke, Beijing, China).

### 2.6. Dual-Luciferase Assays

293T cells in six-well plates were grown to an 80% confluence and transfected with the dual-luciferase reporter plasmid using PEI. At 48 h post-transfection, the cells were harvested and lysed. Debris was removed by centrifugation, after which the luciferase intensity was measured, following the manufacturer’s protocol (Promega, Madison, WI, USA).

### 2.7. Untargeted Metabolomics

Samples were snap-frozen in liquid nitrogen, and 400 μL of extraction solution (methanol/water = 4:1, *v*/*v*) containing 0.02 mg/mL of internal standard (L-2-chlorophenylalanine) was added. Samples were ground by a frozen tissue grinder (Wonbio-96c, Shanghai Wanbo Biotechnology Co., LTD) at 50 Hz for 6 min at −10 °C, followed by ultrasound at 40 kHz for 30 min at 5 °C. The samples were placed at −20 °C for 30 min to facilitate protein precipitation and centrifuged at 13,000× *g* at 4 °C for 15 min, and the supernatant was taken for sample analysis. The quality control sample was prepared by mixing equal volumes of all the samples.

The LC-MS analysis was performed using the Thermo UHPLC-Q Exactive HF-X system equipped with an ACQUITY HSS T3 column (100 mm × 2.1 mm i.d., 1.8 μm; Waters, Milford, MA, USA) at Majorbio Bio-Pharm Technology Co., Ltd. (Shanghai, China). The mobile phases consisted of 0.1% formic acid in water/acetonitrile (95:5, *v*/*v*) (solvent A) and 0.1% formic acid in acetonitrile/isopropanol/water (47.5:47.5, *v*/*v*) (solvent B). The sample injection volume was 3 μL, and the flow rate was set to 0.40 mL/min. The column temperature was maintained at 40 °C. The mass spectrometric data were collected using a Thermo UHPLC-Q Exactive HF-X Mass Spectrometer equipped with an electrospray ionization (ESI) source operating in positive and negative mode. The optimal conditions were set as follows: source temperature at 425 °C; sheath gas flow rate at 50 arb; Aux gas flow rate at 13 arb; ion-spray voltage floating (ISVF) at −3500 V in negative mode and 3500 V in positive mode, respectively; and normalized collision energy, 20-40-60 V rolling for MS/MS. The full MS resolution was 60,000, and the MS/MS resolution was 7500. Data acquisition was performed with the Data-Dependent Acquisition (DDA) mode. The detection was carried out over a mass range of 70–1050 *m/z*.

### 2.8. Metabolomics Data Analysis

The pretreatment of the LC/MS raw data was performed by Progenesis QI v4.2 (Waters Corporation, Milford, CT, USA) software. Metabolites were identified and validated by aligning the molecular mass data (*m*/*z*) using the HMDB (http://www.hmdb.ca/) (accessed on 25 February 2022), Metlin (https://metlin.scripps.edu/) (accessed on 25 February 2022), and the Majorbio Database. The data matrix was then uploaded to the Majorbio cloud platform (https://cloud.majorbio.com) (accessed on 25 February 2022) for data analysis. Metabolic features detected at least 80% in any set of samples were retained. Variables in QC samples with a relative standard deviation (RSD) > 30% were excluded to ensure data reliability. The data were log10-transformed prior to analysis. Sequentially, a principal component analysis (PCA), orthogonal partial least squares discriminant analysis (OPLS-DA), and 7-fold cross-validation were conducted using the “ropls” R package (v1.6.2). The variable importance in the projection (VIP) > 1 obtained by the OPLS-DA model and the *p* < 0.05 generated by Student’s *t*-test were set as the standards to screen the differentially expressed metabolites. The graphs of the PCA, volcano plots, VIP analysis, and HMDB classification were generated by the Majorbio cloud platform. The KEGG pathway enrichment analyses were based on the KEGG database (http://www.genome.jp/kegg/) (accessed on 9 March 2024) and MetaboAnalyst (http://www.metaboanalyst.ca/) (accessed on 9 March 2024).

### 2.9. NAD+ Measurement

NAD+ levels were measured using the NAD+/NADH Assay Kit with WST-8 (S0175, Beyotime, Shanghai, China) according to the manufacturer’s instructions. In detail, transfected RD cells (1 × 10^6^ cells/sample) were collected and lysed by the addition of 200 µL of NAD+/NADH extraction solution. After centrifugation at 12,000× *g* for 10 min at 4 °C, the supernatant was harvested to measure the total NAD+/NADH levels, and 100 µL of supernatant was heated for 30 min at 60 °C to remove NAD+ in order to measure the NADH levels. A 20 µL sample and 90 µL alcohol dehydrogenase were added to a 96-well plate and incubated for 10 min at 37 °C. An amount of 10 µL of chromogenic solution was added and incubated for 30 min at 37 °C. The absorbance at 450 nm was measured using a microplate reader (Thermo Fisher Scientific, Waltham, MA, USA), and the relative NAD+ level was calculated. The amount of NAD+ was derived by subtracting NADH from total NAD+/NADH.

### 2.10. Immunofluorescence

Infected cells were fixed with 4% paraformaldehyde for 20 min, permeabilized with 0.2% triton X-100 (SolarBio, Beijing, China) for 30 min, and blocked with 3% BSA (SolarBio, China) for 30 min. The cells were then incubated with primary antibody for 1.5 h. The primary antibodies were rabbit anti-SIRT1 (1:200, GeneTex, San Antonio, TX, USA), after being washed three times with PBS and then incubated with secondary antibody for 45 min. The secondary antibodies used were Alexa Fluor 488 conjugated goat anti-rabbit IgG (1:1000, Thermo). Cell nuclei were visualized using DAPI staining (SolarBio, China). Fluorescent images were obtained with immunofluorescence microscopy (Nikon Ts2-FL, Tokyo, Japan).

### 2.11. CCK8

Cells were seeded at a density of 5 × 10^3^ cells per well in a 96-well plate and cultured for 24 h. Then they were incubated with different concentrations of NAD+, L-Cystathionine, flavin adenine dinucleotide, L-Acetylcarnitine (hydrochloride), and 8-Hydroxyguanosine as indicated for 48 h. After that, CCK8 (APExBIO, K1018) was added to each well and incubated at 37 °C for 2 h. The absorbance was measured at 450 nm using a microplate reader (Thermo Fisher Scientific, USA).

### 2.12. Mouse Pup Infection Model

One-day-old specific-pathogen-free (SPF) BALB/c neonatal mice (Chongqing Medical University) were used to establish the animal model of viral infection. The neonatal mice were randomly divided into 3 groups: mock, EV-D68-infected, and EV-D68 with NAD+ treatment (n = 6, each group). The EV-D68 (1 × 10^5^ PFU/mouse) or mock (DMEM) were injected into both sides of the ventricles of the pups’ brains with a volume of 40 µL. Then, the neonates were intraperitoneally injected with NAD+ (10 mg/kg) or the control (NaCl) after 6 h infection and received treatment once a day for one week. The survival rates and mean clinical symptoms were daily monitored for 14 days post-infection.

### 2.13. H&E Stain

The pups’ organs (heart, brain, spleen, lung, muscle (hind leg), muscle (spine), spinal cord, and kidney) were obtained for staining. After fixing the tissues in 4% paraformaldehyde for 3 days, the tissues were dehydration-embedded in paraffin. After the embedded tissue was sectioned into 4 μm sections with a micro-chipper and tiled on adhesive glass slides, the slices were incubated overnight at 50 °C. The tissues were stained using Hematoxylin and Eosin (H&E), and the pathological findings were quantified following the criteria for lesions in rats and mice [18].

### 2.14. Statistical Analysis

All the experimental data were analyzed using GraphPad Prism 9.0 (GraphPad Software, San Diego, CA, USA). Quantitative data are presented as the mean ± standard error of the mean (s.e.m.) from at least three independent experiments. Student’s *t*-test was used to compare two groups; a one-way or two-way analysis of variance was followed by Tukey’s post hoc test to compare multiple groups. The probability of survival was determined with the Kaplan–Meier method and compared using the log-rank test. *p* > 0.05 was considered statistically nonsignificant.

## 3. Results

### 3.1. EV-D68 Infection Influences Cellular Metabolism

Viruses are known to alter the host cell metabolism to facilitate their replication, making the identification of virus-induced metabolic reprogramming crucial for understanding host–virus interactions. Here, we conducted an untargeted metabolomic analysis to assess alterations in host metabolites following EV-D68 infection.

**Figure 1:**
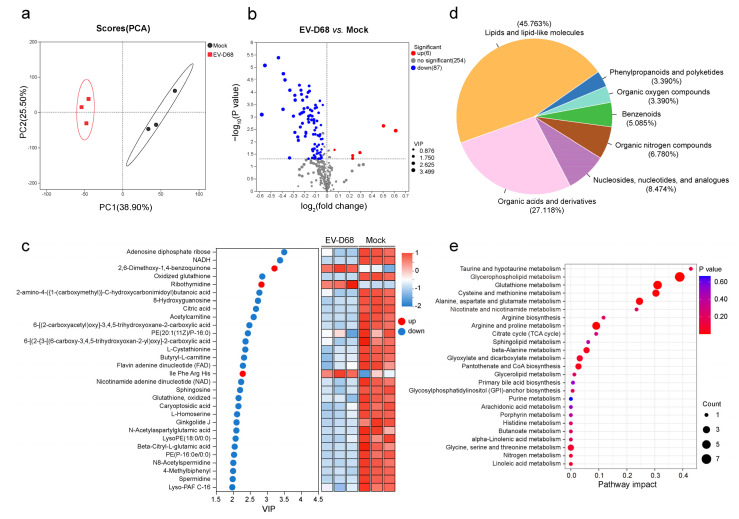
EV-D68 infection influences cellular metabolism. RD cells cultured in 10 cm dishes infected with EV-D68 Fermon at an MOI of 1 and non-infected as control. After 18 h infection, cells were harvested for LC-MS analysis (n = 3 samples per group). (**a**) Principal component analysis of metabolomics data in the mock and EV-D68-infected group. (**b**) Volcano map of differentially expressed metabolites in EV-D68 compared with that in the mock group. (**c**) VIP analysis of the top 30 differentially expressed metabolites between the mock and EV-D68 group. The color of each rectangle in the right panel represents the relative level of the metabolites. The metabolites with VIP > 1 and *p* < 0.05 were determined as significantly different metabolites. The full name of metabolites were shown in in Supplementary Tables S1–S4. (**d**) Pie charts showing the HMDB classifications of the 93 differential metabolites. (**e**) KEGG pathway enrichment analysis of the significantly altered metabolites between the mock and EV-D68 group.

Principal component analysis (PCA) revealed a clear separation of metabolite profiles between mock-infected and EV-D68-infected cells (Figure 1a). Using an orthogonal partial least squares discriminant analysis (OPLS-DA), we identified differential metabolites, with VIP > 1 and *p* < 0.05 as the screening criteria. EV-D68 infection resulted in significant changes in 93 metabolites, with 87 downregulated and 6 upregulated (Figure 1b). VIP analysis showed the top 30 differential metabolites after EV-D68 infection and some of the downregulated metabolites participated in the redox reactions, which may indicate the significance of redox balance during EV-D68 infection (Figure 1c). For example, NAD(H) and flavin adenine dinucleotide (FAD), essential cofactors in metabolic redox reactions, play critical roles in the TCA cycle and the biosynthesis of lipids, nucleotides, and amino acids. Other metabolites, such as glutathione, citric acid, L-Cystathionine, acetylcarnitine, and 8-Hydroxyguanosine, also directly or indirectly influence cellular redox homeostasis. Further analysis categorized the differential metabolites using the Human Metabolome Database (HMDB). Over 80% of the altered metabolites belonged to the categories “Lipids and lipid-like molecules”, “Organic acids and derivatives”, and “Nucleosides, nucleotides, and analogues” (Figure 1d). To pinpoint the key metabolic pathways affected by EV-D68 infection, we performed a KEGG pathway enrichment analysis. The results showed significant enrichment in pathways including taurine and hypotaurine metabolism, glycerophospholipid metabolism, glutathione metabolism, cysteine and methionine metabolism, alanine, aspartate, and glutamate metabolism, and nicotinate and nicotinamide metabolism (Figure 1e). Taken together, these findings indicate that EV-D68 infection induces extensive metabolic reprogramming.

### 3.2. NAD+ Suppresses EV-D68 Proliferation In Vitro

**Figure 2:**
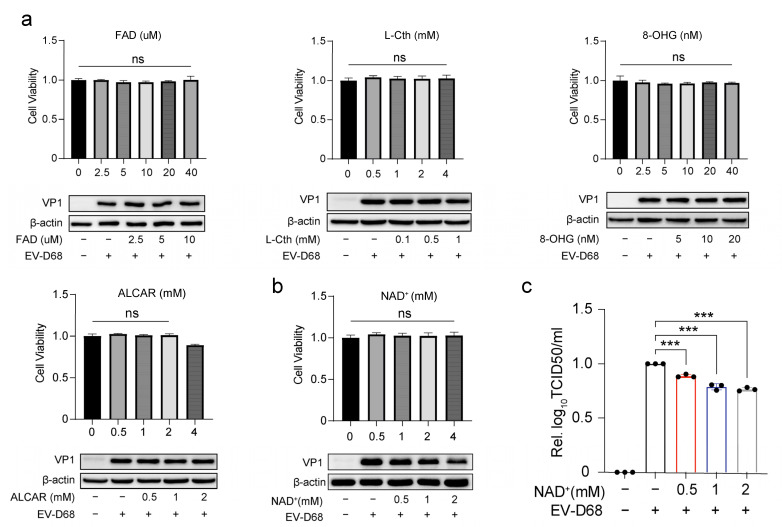
NAD+ suppresses EV-D68 proliferation in vitro. (**a**,**b**) RD cells were incubated with different concentrations of flavin adenine dinucleotide (FAD), L-Cystathionine (L-Cth), 8-Hydroxyguanosine (8-OHG), L-Acetylcarnitine (ALCAR), or nicotinamide adenine dinucleotide (NAD+) as indicated for 48 h. Then the cell viability was assessed by Cell Counting Kit-8 (CCK-8) assay. RD cells were treated with different concentrations of exogenous FAD (0, 2.5, 5, and 10 µM), 8-OHG (0, 5, 10, and 20 nM), L-Cth (0, 0.1, 0.5, and 1 mM), ALCAR (0, 0.5, 1, and 2 mM), and NAD+ (0, 0.5, 1, and 2 mM) 6 h prior to infection with EV-D68 Fermon at an MOI of 1 for 18 h. Viral VP1 protein levels were assessed by Western blot. (**c**) As with the cell line and experiment mentioned above, RD cells were treated with different concentrations of exogenous NAD+ (0, 0.5, 1, and 2 mM) 6 h prior to infection with EV-D68 Fermon at an MOI of 1, and after 24 h the supernatant of the cells was collected. Viral titers were determined using TCID50. Data in all quantitative panels are normalized based on β-actin presented as the mean ± SD of n = 3 replicates. Error bars indicate SD (n = 3). “−” represents absence, and “+” represents presence. *** *p* < 0.001; ns, not significant.

To investigate the anti-EV-D68 effects of the significantly altered metabolites identified above, we selected five downregulated compounds—nicotinamide adenine dinucleotide (NAD+), acetylcarnitine (ALCAR), L-Cystathionine (L-Cth), flavin adenine dinucleotide (FAD), and 8-Hydroxyguanosine (8-OHG)—for further validation. RD cells were treated with different concentrations of these compounds or mock-treated and subsequently infected with EV-D68. Our results revealed that only NAD+ significantly inhibited viral protein expression, as evidenced by a dose-dependent reduction in VP1 protein levels with increasing concentrations of exogenous NAD+ (0.5 mM to 2 mM). A cell viability assay confirmed that NAD+ exhibited no significant cytotoxicity at concentrations up to 4 mM. In contrast, the other compounds showed no notable inhibitory effects on EV-D68 replication at non-cytotoxic concentrations (Figure 2a,b). Furthermore, viral titers decreased significantly following NAD+ treatment (Figure 2c). These findings suggest that NAD+ possesses potential antiviral activity against EV-D68.

### 3.3. NAD+ Suppresses EV-D68 Proliferation In Vivo

**Figure 3:**
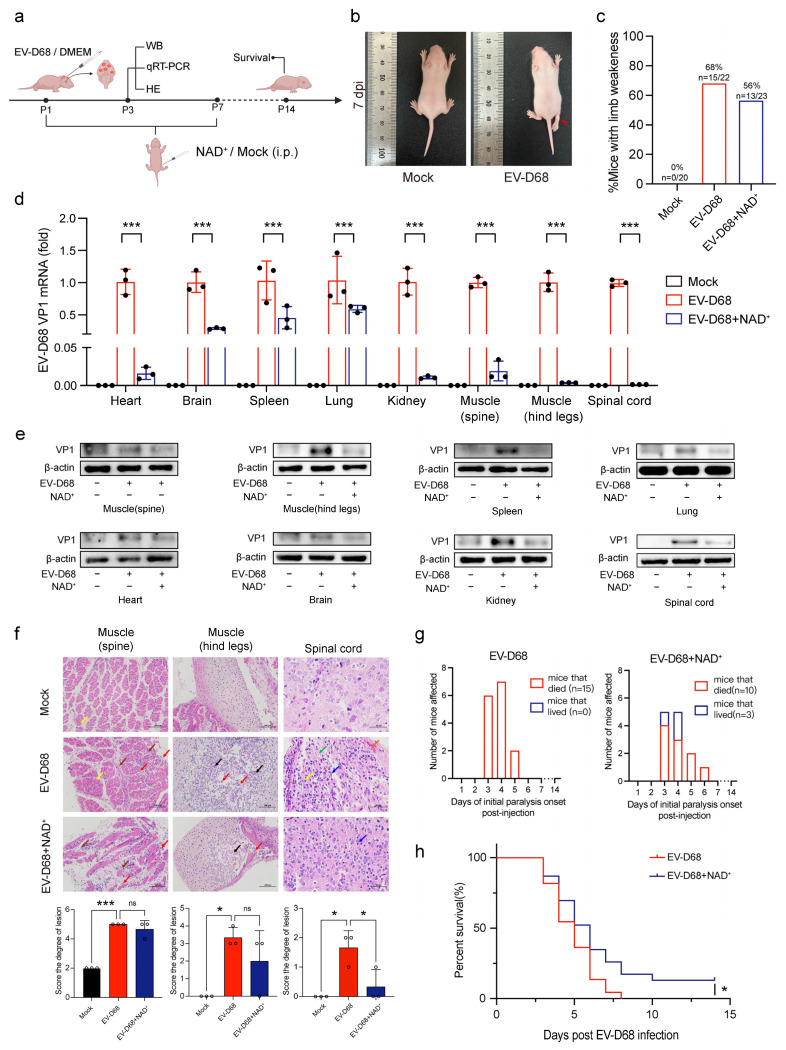
NAD+ suppresses EV-D68 proliferation in vivo. (**a**) Schematic representation of the experimental design for NAD+ treatment in neonatal mice infected with EV-D68(ATCC VR-1824). Mice were intracranially inoculated with EV-D68 (1 × 10^5^ PFU/mouse) and treated with continuous NAD+ therapy until the seventh day. Viral loads in major organs and tissues were evaluated using RT-qPCR on the 3rd day, and survival rates were monitored until the 14th day. (**b**) Representative images showing symptoms of healthy mice in the mock group and the mice with limb paralysis in the EV-D68(ATCC VR-1824) infection group on 7 dpi. (**c**) Bar graph showing the percentage of mice with paralysis between the EV-D68-infected and NAD+ treatment groups. (**d**,**e**) Levels of EV-D68 viral nucleic acids and VP1 protein in major organs and muscle tissues were quantified using Western blot and RT-qPCR on the third day. (**f**) Histopathological changes in tissues from mock, EV-D68-infected, and EV-D68-infected with NAD+ treatment groups (n = 3) were analyzed. Pathological images of spine muscles and hind limb muscles, scale bar = 100 μm. Yellow arrow: Vacuolar degeneration of muscle cells. Brown arrow: Fibrosis of interstitial fibrous connective tissue. Red arrow: Infiltration of lymphocytes and granulocytes. Black arrow: Necrosis of chondrocytes. Pathological images of the spinal cord, scale bar = 50 μm. Blue arrow: Atrophied neurons. Yellow arrow: Proliferation of gliosis. Green arrow: Neuronal necrosis. Orange arrow: Vascular congestion. (**g**) Day of paralysis onset in mice after injection with EV-D68(ATCC VR-1824). (**h**) Survival rate statistics for the NAD+ treatment (n = 23) and saline control groups (n = 22) following EV-D68 infection were compared. Data in all quantitative panels are normalized based on β-actin or GAPDH presented as the mean ± SD of n = 3 replicates. Error bars indicate SD (n = 3). “−” represents absence, and “+” represents presence. * *p* < 0.05; *** *p* < 0.001; ns, not significant.

To evaluate the antiviral effect of NAD+ in vivo, we used a neonatal mouse model of EV-D68 infection. Mice less than one day old were intracranially injected with EV-D68, followed by the intraperitoneal administration of NAD+. NAD+ therapy was continued daily for seven days. On day three post-infection, viral loads in various tissues were measured, and histopathological changes were assessed. Survival rates were recorded from day 1 to day 14 post-infection (Figure 3a). After 7 days, we observed hind limb paralysis in the pups, which was likely due to nerve damage associated with acute flaccid myelitis (AFM) caused by EV-D68 infection (Figure 3b). Compared to the EV-D68-infected group, the NAD+ treatment group had fewer signs of morbidity (Figure 3c). Furthermore, our results showed that, compared to the EV-D68 infection group (n = 22), mice treated with NAD+ (n = 23) exhibited significantly lower levels of viral nucleic acids and VP1 protein in the heart, brain, spleen, lungs, kidneys, spinal muscles, and hind limb muscles (Figure 3d,e). HE staining analysis revealed that EV-D68 infection significantly exacerbated pathological tissue damage. In muscle tissues, EV-D68 infection led to pathological changes such as the vacuolar degeneration of muscle cells, cartilage necrosis, and prominent lymphocyte infiltration. In the NAD+ treatment group, lesion scores were reduced, although the difference did not reach statistical significance, potentially due to the limited duration of the NAD+ treatment. In spinal cord tissues, EV-D68 infection induced neuronal shrinkage, necrosis, and glial cell proliferation, whereas in the NAD+ treatment group, only minor neuronal shrinkage and alleviated pathological damage were observed (Figure 3f). Moreover, no significant pathological changes were observed in other tissues. This may be due to the short infection duration or the tissue-specific tropism of EV-D68. These findings suggest that NAD+ treatment partially mitigates the pathological damage caused by EV-D68 infection, particularly demonstrating significant protective effects in spinal cord tissue. By comparing the days of initial paralysis from both groups, the EV-D68 with NAD+ treatment group showed a more dispersed onset of paralysis, extending from day 3 to day 6 (Figure 3g). Infected mice which were injected with NAD+ also showed higher survival rates, with three mice surviving beyond 14 days, accounting for a survival rate of 13%, while all mice in the control group had died at 8 days post-infection (Figure 3h). These results suggest that supplementation with NAD+ may confer a protective effect against the paralysis and mortality induced by EV-D68 infection in mice, as evidenced by the increased survival rate and reduced number of mortalities. Collectively, the results above suggest that NAD+ suppresses virus proliferation both in vivo and in vitro.

### 3.4. Viral Infection Regulated the Expression of Enzymes in the NAD+ Salvage Pathway, Leading to a Reduction in NAD+ Level

**Figure 4:**
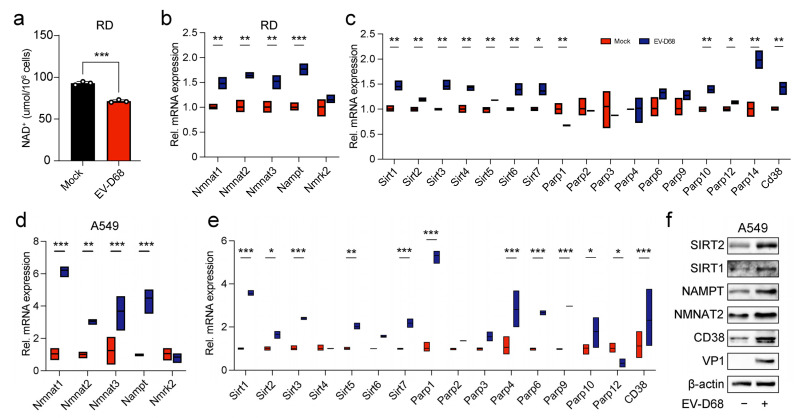
Viral infection regulated the expression of enzymes in the NAD+ salvage pathway, leading to a reduction in NAD+ level. (**a**) RD cells were cultured in 6-well plates and infected with EV-D68 Fermon at an MOI of 0 or 1 for 24 h. WST—detection of NAD+ in cells by WST-8 reaction colorimetry. (**b**–**e**) RD and A549 cells were infected with EV-D68 Fermon at an MOI of 1. Then, 18 h later, the expression changes of NAD+ synthesis genes (Nmnat1 to 3, Nampt, and Nmrk2) and NAD+ consuming genes (Sirt1 to 7, Parp1 to 6, Parp9, 10, 12, 14, and CD38) were measured using RT-qPCR. (**f**) A549 cells were infected with EV-D68 Fermon at an MOI of 1. Then, 18 h later, the protein levels of VP1, SIRT1, SIRT2, NAMPT, NMNAT2, and CD38, compared to the control β-actin, were determined using Western blot. Data in all quantitative panels are normalized based on β-actin or GAPDH presented as the mean ± SD of n = 3 replicates. Error bars indicate SD (n = 3). “−” represents absence, and “+” represents presence. * *p* < 0.05; ** *p* < 0.01; *** *p* < 0.001.

Given that NAD+ levels were downregulated following EV-D68 infection, as indicated by the metabolomics analysis, and NAD+ exhibited antiviral activity both in vivo and in vitro, we further quantified NAD+ levels in EV-D68-infected RD cells (Figure 4a). The results showed a significant decline in NAD+ levels after infection. To investigate the underlying mechanism, we assessed the expression of genes involved in NAD+ synthesis and consumption using RT-qPCR. The results revealed that, upon viral infection, the mRNA levels of NAD+-consuming enzymes, including Sirt1-7, Parp1, Parp10, Parp12, Parp14, and CD38, were substantially upregulated. Similarly, the NAD+-synthesizing enzymes Nmnat1–3 and Nampt also showed an increased expression (Figure 4b,c). These findings suggest that the reduction in NAD+ levels during EV-D68 infection may result from an imbalance, where NAD+-consuming enzymes outweigh the activity of NAD+-synthesizing enzymes. The results in A549 cells demonstrated that most NAD+ synthesis and consumption genes were significantly upregulated at the mRNA level as well (Figure 4d,e). Based on these findings, we selected five genes—Nampt, Nmnat2, Sirt1, Sirt2, and CD38—that were consistently upregulated in both RD and A549 cells, to evaluate their protein expression levels after viral infection (Figure 4f). The results confirmed that the protein levels of these genes increased in parallel with their mRNA levels. Collectively, our data highlight the significant alterations in NAD+ metabolism during EV-D68 infection, likely driven by the dysregulation of metabolic enzymes within the NAD+ pathway.

### 3.5. NAD+-Consuming Enzyme SIRT1 Inhibits EV-D68 Replication

**Figure 5:**
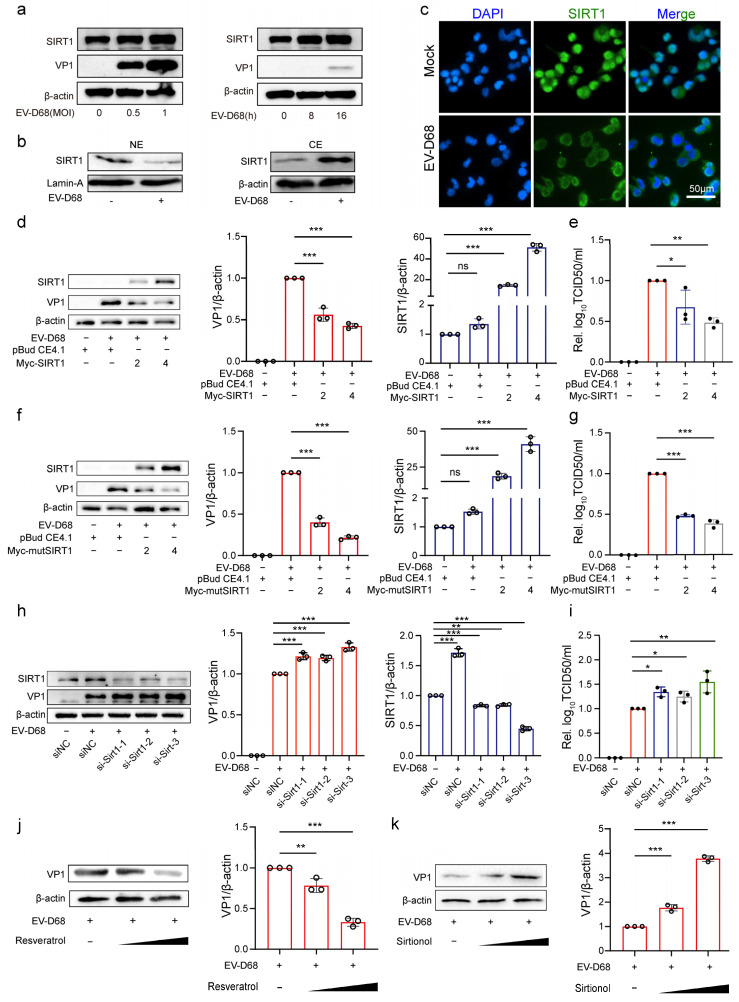
NAD+-consuming enzyme SIRT1 inhibits EV-D68 replication. (**a**) A549 cells were infected with EV-D68 Fermon at a multiplicity of infection (MOI) of 0, 0.5, and 1. Samples were collected after 24 h. Another group were infected with EV-D68 Fermon at an MOI of 1; samples were collected at 0, 8, and 16 h post-infection (hpi). Protein levels of SIRT1 and VP1 were detected using Western blot. (**b**) RD cells infected with EV-D68 Fermon at an MOI of 1 for 24 h. Cytoplasm extracts (CEs) and nuclear extracts (NEs) were prepared. SIRT1, β-actin, and lamin A expression levels were detected by Western blot analyses using the corresponding antibodies. (**c**) RD cells were infected with EV-D68 Fermon at an MOI of 1, and SIRT1 localization was assessed by immunofluorescence at 24 hpi. The SIRT1 was stained with the preliminary antibody rabbit anti-SIRT1 and secondary florescent affiliate antibody goat anti-rabbit AlexaFluor488. The nuclei were counterstained with DAPI. Scale bar represents 50 μm. (**d**–**g**) 293T cells were transfected with pBudCE4.1-Sirt1 or pBudCE4.1-mtSirt1 at 2, 4 μg, with pBudCE4.1 as the control. After 48 h, cells were infected with EV-D68 Fermon at an MOI of 1 for 24 h. Protein levels of SIRT1 and viral VP1 were quantified by Western blot. The intensity of Western blot band signals was quantified behind by Image J 1.54g (**d**,**f**). And viral titers were measured using TCID50 (**e**,**g**). (**h**,**i**) 293T cells were transfected with si-Sirt1-1, si-Sirt1-2, and si-SIRT1-3 (30 nM), with si-NC as the control, for 48 h. After that, cells were infected with EV-D68 Fermon at an MOI of 1 for 24 h. Protein levels of SIRT1 and viral VP1 were quantified by Western blot. The intensity of Western blot band signals was quantified behind by Image J (**h**). And viral titers were measured using TCID50 (**i**). (**j**,**k**) RD cells were treated with Resveratrol and Sirtinol in different doses (0, 20, 40 µM). After 8 h, cells were infected with EV-D68 Fermon for 24 h at an MOI of 1. Protein levels of viral VP1 were quantified by Western blot. The intensity of Western blot band signals was quantified behind by Image J. Data in all quantitative panels are normalized based on β-actin or GAPDH presented as the mean ± SD of n = 3 replicates. Error bars indicate SD (n = 3). “−” represents absence, and “+” represents presence. * *p* < 0.05; ** *p* < 0.01; *** *p* < 0.001; ns, not significant.

Considering our results and the demonstrated antiviral effects of SIRT1 against multiple viruses, we focused on the role of SIRT1 during EV-D68 infection. The results showed that SIRT1 expression increased proportionally with the multiplicity of infection (MOI) and the duration of post-infection time (Figure 5a), indicating that EV-D68 infection induces SIRT1 expression. Immunofluorescence results showed that EV-D68 infection induced SIRT1 subcellular translocation from the nucleus to the cytoplasm (Figure 5b,c).

Given the observed upregulation and translocation of SIRT1, we next evaluated its effect on viral replication. Plasmids expressing SIRT1 (pBudCE4.1-SIRT1) or a control plasmid (pBudCE4.1) were transfected into 293T cells, followed by infection with EV-D68. The results showed that the overexpression of SIRT1 significantly reduced EV-D68 VP1 protein expression and decreased viral titers compared with the control group (Figure 5d,e). After that, we constructed a mutant SIRT1 plasmid (pBudCE4.1-mtSIRT1) lacking the nuclear localization signal (NLS). Interestingly, the mutant SIRT1 exhibited similar antiviral effects to wild-type SIRT1 (Figure 5f,g), indicating that SIRT1 inhibited viral replication independent of its transcriptional activity.

Furthermore, the siRNA-mediated knockdown of SIRT1 was performed following EV-D68 infection. The knockdown of SIRT1 significantly increased EV-D68 VP1 protein expression and viral titers compared to the si-NC group (Figure 5h,i). Additionally, the effects of SIRT1 modulation were assessed using the SIRT1 agonist Resveratrol and the inhibitor Sirtinol. Resveratrol treatment reduced VP1 expression in a dose-dependent manner (Figure 5j), while Sirtinol treatment had the opposite effect, increasing VP1 expression (Figure 5k). These findings collectively demonstrate that SIRT1 functions as an inhibitor of EV-D68 replication.

### 3.6. NAD+ Augments the Antiviral Effect of SIRT1

**Figure 6:**
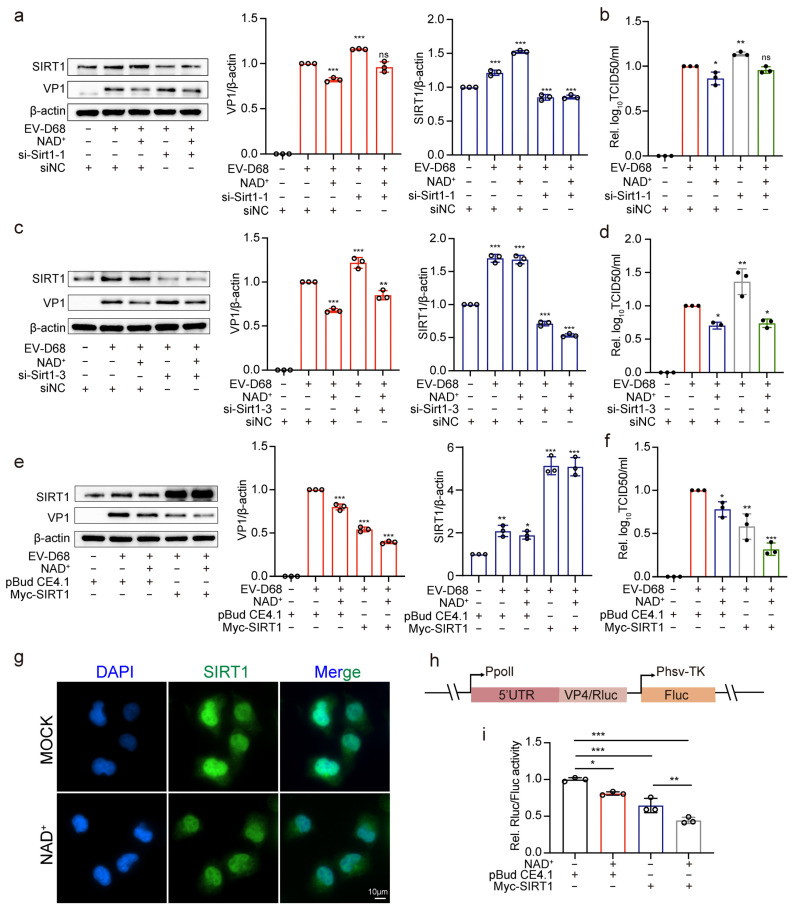
NAD+ augments the antiviral effect of SIRT1. (**a**,**b**) 293T cells were transfected with si-Sirt1-1 and si-NC for 30 nM; 48 h later, cells were treated with NAD+ for 8 h and then infected with EV-D68 Fermon at an MOI of 1 for 24 h. Protein levels of SIRT1 and viral VP1 were detected by Western blot. The intensity of Western blot band signals was quantified behind by Image J (**a**). And viral titers were measured using TCID50 (**b**). (**c**,**d**) 293T cells were transfected with si-Sirt1-3 and si-NC for 30 nM. Then, 48 h later, followed the same experiment mentioned before (**a**,**b**). (**e**,**f**) 293T cells were transfected with pBudCE4.1-Sirt1 and pBudCE4.1 for 2 μg. Then, followed the same experiment mentioned before (**a**,**b**). (**g**) RD cells were treated with NAD+ for 8 h, and SIRT1 localization was assessed by immunofluorescence. The SIRT1 was stained with the preliminary antibody rabbit anti-SIRT1 and secondary florescent affiliate antibody goat anti-rabbit AlexaFluor488. Thr nuclei were counterstained with DAPI. Scale bar represents 10 μm. (**h**) Schematic presentation of the luciferase reporter plasmid, containing a pol I promoter, the 5′ UTR region of EV-D68, and the Renilla luciferase gene (Rluc) infused with the first 15 bps of EV-D68 ORF, and a Firefly luciferase reporter (Fluc) gene driven by TK promoter. (**i**) Cells were co-transfected with p5Ferm and either pBudCE4.1-SIRT1 or the control vector. After 24 h, cells were treated with NAD+ for 8 h. Cells were lysed and their Rluc and Fluc activities measured. Determining the ratio of Rluc activity to Fluc activity yielded the relative IRES activity. Data in all quantitative panels are normalized based on β-actin or GAPDH presented as the mean ± SD of n = 3 replicates. Error bars indicate SD (n = 3). “−” represents absence, and “+” represents presence. * *p* < 0.05; ** *p* < 0.01; *** *p* < 0.001; ns, not significant.

Since NAD+ is a substrate of SIRT1, we investigated the role of NAD+ in the antiviral effects of SIRT1. The siRNA-mediated knockdown of SIRT1 (si-Sirt1) led to a significant increase in viral replication. However, this effect was mitigated by NAD+ supplementation, which suppressed viral replication and reduced viral titers in the si-Sirt1-treated groups (Figure 6a–d). Conversely, 293T cells were transfected with pBudCE4.1-SIRT1 or the control vector pBudCE4.1. After 48 h, the cells were treated with NAD+ for 8 h, followed by infection with EV-D68. The results showed that the addition of NAD+ significantly enhanced the antiviral effect of SIRT1, as evidenced by reduced viral replication and viral titers compared to cells overexpressing SIRT1 alone (Figure 6e,f). These findings suggest that NAD+ enhances the antiviral activity of SIRT1.

Considering that SIRT1 exerts antiviral effects by exiting the nucleus, immunofluorescence results show that the exogenous addition of NAD+ did not affect the subcellular localization of SIRT1 (Figure 6g). Therefore, to investigate the mechanism of NAD+ in augmenting the antiviral effect of SIRT1, we constructed an EV-D68 5′UTR dual-luciferase reporter plasmid, p5Ferm (Figure 6h) [17]. 293T cells were co-transfected with p5Ferm and either pBudCE4.1-SIRT1 or the control vector. The exogenous addition of NAD+ and the overexpression of SIRT1 both significantly suppressed the luciferase activity driven by the EV-D68 5′UTR, suggesting that NAD+ and SIRT1 inhibit EV-D68 5′UTR-mediated translation. Notably, the overexpression of SIRT1 with the addition of NAD+ further strengthened this inhibitory effect (Figure 6i), underscoring its ability to enhance the antiviral action of SIRT1.These findings demonstrate that NAD+ synergistically enhances the antiviral effects of SIRT1 by suppressing EV-D68 replication and inhibiting 5′UTR-mediated translation.

## 4. Discussion

NAD+ functions as a critical coenzyme and co-substrate for enzymes involved in a wide range of physiological and pathological processes, including cancer, aging, and neurodegeneration [19,20,21,22,23,24]. Our metabolomics analysis revealed a significant reduction in NAD+ levels following EV-D68 infection, suggesting that viral infection induces metabolic disturbances. Similarly, a study by Pang et al. reported a decline in NAD+ levels during Zika virus (ZIKV) infection [10]. Nevertheless, the specific molecular mechanisms underlying the decrease in NAD+ were not delineated. Our findings indicate that although both NAD+ synthesis and consumption genes were upregulated upon EV-D68 infection, the rate of NAD+ synthesis may not compensate for the elevated consumption, ultimately resulting in decreased NAD+ levels. Interestingly, previous studies have reported that the NAD+ synthesis or consumption genes NAMPT, SIRT1, SIRT2, and CD38 were upregulated during various viral infections [25,26,27,28], while Nmnat2 was found to decrease following Zika virus infection [10].

Additionally, previous research demonstrated that NMN, a precursor of NAD+, can protect 30% of aged mice from lethal SARS-CoV-2 infection [29]. However, the antiviral mechanisms of NAD+ remain incompletely understood. NAD+-consuming enzymes, such as PARPs and Sirtuins, have been reported to exhibit antiviral functions, with NAD+ serving as a necessary substrate for their activity [12,30]. These observations highlight that genes related to NAD+ metabolism plays a role in antiviral immunity.

For the NAD+ consumers, the sirtuin (SIRT) protein family comprises seven members (SIRT1–SIRT7), each playing diverse roles in various viral infections [31]. Specifically, SIRT1, SIRT2 and SIRT5 have been shown to exhibit potent pro-viral activity by facilitating the replication of a range of viral pathogens [32,33,34,35], while in certain viral infections, SIRT1, SIRT5, and SIRT6 display anti-viral properties [36,37,38]. Our study found that EV-D68 can induces SIRT1 expression, which consequently inhibits viral replication, similar to the effects observed during EV71 infection [39], highlighting its potential as a therapeutic target for combating EV-D68 infection.

Our study further demonstrated that NAD+ treatment enhanced the antiviral activity of SIRT1. Sirtuins, including SIRT1, utilize NAD+ as a cofactor to facilitate the binding and deacetylation of specific target proteins, leading to effects such as promoting DNA repair, regulating inflammation, and exhibiting antioxidant properties [40]. As an NAD+-dependent protein deacetylase, the activity of SIRT1 is largely influenced by the intracellular NAD+/NADH ratio. An increase in the NAD+/NADH ratio enhances SIRT1 activity [41]. Our findings underscore the role of NAD+ in enhancing SIRT1′s antiviral effects.

Furthermore, our results showed that SIRT1 inhibited the expression of reporter genes controlled by the IRES in the 5′UTR region of EV-D68 in reporter plasmids. These findings are consistent with an earlier report that SIRT1 binds to the IRES of the EV-A71 5′UTR to attenuate viral RNA translation [39]. As an RNA virus, EV-D68 replicates primarily in the cytoplasm. Notably, we observed that SIRT1 translocated from the nucleus to the cytoplasm in EV-D68-infected cells. The overexpression of both wild-type and mutant SIRT1 significantly enhanced the antiviral capabilities of cells, suggesting that SIRT1 may inhibit the IRES of EV-D68 5′UTR through nucleocytoplasmic shuttling. Additionally, studies have reported that SIRT1 regulates the transcriptional factor REST, which has been shown to bind the REST1/NRSE sequence between the promoter of HSV-1 immediate early genes ICP4 and ICP22, thereby inhibiting the transcription of these genes. It can be speculated that SIRT1, by regulating REST, may in turn affect the regulation of HSV-1 lytic cycle genes by REST. Therefore, another mechanism by which SIRT1 inhibits enterovirus replication may be through regulating the Repressor Element of viral promoter transcription [42].

The role of SIRT1 in viral infection is complex. In our study, we observed a significant upregulation of SIRT1 levels during EV-D68 infection. Regardless of whether wild-type Sirt1 or mtNLS-Sirt1 was supplemented, both demonstrated robust antiviral activity. Previous studies on EV-A71 infection have reported a decrease in SIRT1 levels, accompanied by an increase in reactive oxygen species (ROS) [39]. Conversely, other findings suggested that SIRT1 expression is upregulated following infection, and supplementation with large amounts of mtNLS-Sirt1 significantly inhibits viral replication [40]. These conflicting observations highlight the complexity of SIRT1’s role in EV-A71 infection. In other studies, the early stage of EV-D68 infection indicates no significant change in SIRT1 levels, with only minimal nuclear translocation observed, while the transfection of wild-type Sirt1 appeared to enhance viral titer. We proposed that the initially low cytoplasmic SIRT1 levels during early infection may limit its ability to counteract viral replication, potentially facilitating viral processes such as vesicular release [41]. However, as SIRT1 levels increase, ROS levels are reduced, which may contribute to enhanced antiviral effects. Moreover, SIRT1 is involved in the regulation of multiple signaling pathways [43,44], though the precise mechanism by which SIRT1 activates or modulates specific pathways in response to EV-D68 infection requires further exploration.

**Figure 7:**
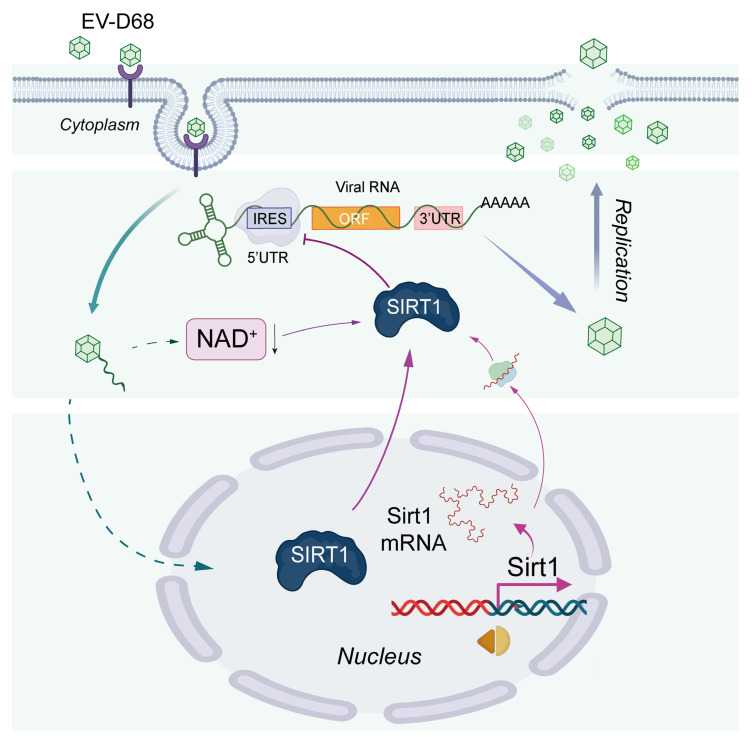
Schematic diagram illustrating the regulatory mechanism of NAD+ and SIRT1 during EV-D68 infection. EV-D68 infection decreases the level of cellular NAD+ (Black arrow: decrease), which promotes the expression of SIRT1. SIRT1 has an inhibitory effect on EV-D68, and it is strengthened by NAD+.

In conclusion, our study revealed that NAD+ treatment effectively suppresses EV-D68 infection both in vivo and in vitro. Additionally, the protective effect of SIRT1 appears to be linked to its enhanced activity, facilitated by the consumption of NAD+. These findings suggest that NAD+ holds promise as a potential therapeutic strategy for combating EV-D68 infection (Figure 7).

## Data Availability

All data referenced in this study are included in the article and Supplementary Materials. Further inquiries can be directed to the corresponding author.

## Supplementary Materials

The following supporting information can be downloaded at: https://www.mdpi.com/article/10.3390/v17020175/s1; Table S1: Primes designed for qRT-PCR. Table S2: Primes designed for siRNA. Table S3: Primes designed for PCR. Table S4: Raw data of metabolites.
